# Supplementary material for: Clinical acuity and National Early Warning Scores (NEWS2) of remotely monitored patients on virtual wards: A retrospective cohort study
Source: PLoS One. 2026 Apr 28;21(4):e0347678. doi: 10.1371/journal.pone.0347678 (PMC13123966; doi:10.1371/journal.pone.0347678)
Supplement: S1 Appendix — (DOCX) [file pone.0347678.s001.docx]

**Table S1: Presence of Doccla recorded clinical observations by patient demographics**

|  | | **N** | **No clinical observations recorded**  **(n=1,746)** | | **Clinical observations**  **recorded**  **(n=2,533)** | |
| --- | --- | --- | --- | --- | --- | --- |
|  | |  | **n** | **%** | **n** | **%** |
| **Age (median, IQR):** | | 4.279 | 82 | (74, 89) | 73 | (60, 82) |
|  | 16-39 years | 244 | 60 | 24.6% | 184 | 75.4% |
|  | 40-49 years | 208 | 44 | 21.2% | 164 | 78.8% |
|  | 50-59 years | 352 | 72 | 20.5% | 280 | 79.5% |
|  | 60-69 years | 582 | 150 | 25.8% | 432 | 74.2% |
|  | 70-79 years | 1,025 | 348/ | 34.0% | 677 | 66.0% |
|  | 80+years | 1,868 | 1,072 | 57.4% | 796 | 42.6% |
| **Sex:** | |  |  |  |  |  |
|  | Male | 1,932 | 790 | 40.9% | 1,142 | 59.1% |
|  | Female | 2,347 | 956 | 40.7% | 1,391 | 59.3% |
| **Source of referral:** | |  |  |  |  |  |
|  | Acute Hospital | 3,489 | 1,287 | 36.9% | 2,202 | 63.1% |
|  | Ambulance Service | 61 | 27 | 44.3% | 34 | 55.7% |
|  | Community Health Service | 447 | 271 | 60.6% | 176 | 39.4% |
|  | Emergency Care Department | 3 | 2 | 66.7% | 1 | 33.3% |
|  | GP Practice | 273 | 154 | 56.4% | 119 | 43.6% |
|  | Not known | 2 | 1 | 50% | 1 | 50% |
|  | Other | 4 | 4 | 100% | 0 | 0% |
| **Pathway*:** | |  |  |  |  |  |
|  | Respiratory | 1,350 | 310 | 23.0% | 1,040 | 77.0% |
|  | Frailty | 1,379 | 933 | 67.7% | 446 | 32.3% |
|  | OPAT | 580 | 97 | 16.7% | 483 | 83.3% |
|  | Heart Failure | 188 | 10 | 5.3% | 178 | 94.7% |
|  | General | 898 | 445 | 49.6% | 453 | 50.4% |
| **Reason for discharge:** | |  |  |  |  |  |
|  | Virtual ward care complete | 3,853 | 1,506 | 39.1% | 2,347 | 60.9% |
|  | Admitted to hospital | 401 | 224 | 55.9% | 177 | 44.1% |
|  | Patient died | 25 | 16 | 64.0% | 9 | 36.0% |
| **Virtual ward length of stay**  **(days; median, IQR)** | | 4.279 | 6 | (3, 10) | 10 | (6, 14) |

*Patients may be on more than one pathway during the same admission.

Note. Percentages are row percentages rather than column percentages.

**Table S2: NEWS2 values and component scores**

|  | | **First**  **(n=2,533)** | | **Maximum**  **(n=2,533)** | | **Deteriorated by**  **(n=2,331)** | |
| --- | --- | --- | --- | --- | --- | --- | --- |
|  | | **n** | **%** | **n** | **%** | **n** | **%** |
| NEWS2 values | |  |  |  |  |  |  |
|  | 0 | 600/2479 | 24.2% | 115/2479 | 4.6% | 696/2331 | 29.9% |
|  | 1-2 | 1051/2479 | 42.4% | 685/2479 | 27.6% | 964/2331 | 41.4% |
|  | 3-4 | 569/2479 | 23.0% | 887/2479 | 35.8% | 513/2331 | 22.0% |
|  | 5-6 | 204/2479 | 8.2% | 568/2479 | 22.9% | 141/2331 | 6.0% |
|  | 7+ | 55/2479 | 2.2% | 224/2479 | 9.0% | 17/2331 | 0.7% |
| Respiratory rate | |  |  |  |  |  |  |
|  | 0 | 2008/2511 | 80.0% | 885/2511 | 35.2% |  |  |
|  | 1 | 68/2511 | 2.7% | 294/2511 | 11.7% |  |  |
|  | 2 | 324/2511 | 12.9% | 798/2511 | 31.8% |  |  |
|  | 3 | 111/2511 | 4.4% | 534/2511 | 21.3% |  |  |
| Oxygen saturation | |  |  |  |  |  |  |
|  | 0 | 1357/2528 | 53.7% | 640/2528 | 25.3% |  |  |
|  | 1 | 584/2528 | 23.1% | 691/2528 | 27.3% |  |  |
|  | 2 | 284/2528 | 11.2% | 418/2528 | 16.5% |  |  |
|  | 3 | 303/2528 | 12.0% | 779/2528 | 30.8% |  |  |
| Systolic blood pressure | |  |  |  |  |  |  |
|  | 0 | 1994/2511 | 79.4% | 1246/2511 | 49.6% |  |  |
|  | 1 | 337/2511 | 13.4% | 643/2511 | 25.6% |  |  |
|  | 2 | 140/2511 | 5.6% | 414/2511 | 16.5% |  |  |
|  | 3 | 40/2511 | 1.6% | 208/2511 | 8.3% |  |  |
| Pulse rate | |  |  |  |  |  |  |
|  | 0 | 1875/2521 | 74.4% | 1096/2521 | 43.5% |  |  |
|  | 1 | 569/2521 | 22.6% | 1101/2521 | 43.7% |  |  |
|  | 2 | 68/2521 | 2.7% | 256/2521 | 10.2% |  |  |
|  | 3 | 9/2521 | 0.4% | 68/2521 | 2.7% |  |  |
| Temperature | |  |  |  |  |  |  |
|  | 0 | 2054/2516 | 81.6% | 1156/2516 | 45.9% |  |  |
|  | 1 | 428/2516 | 17.0% | 1168/2516 | 46.4% |  |  |
|  | 2 | 6/2516 | 0.2% | 13/2516 | 0.5% |  |  |
|  | 3 | 28/2516 | 1.1% | 179/2516 | 7.1% |  |  |
| Doccla recorded NEWS2 values | |  |  |  |  |  |  |
|  | 0 | 267/982 | 27.2% | 106/982 | 10.8% | 257/728 | 35.3% |
|  | 1-2 | 432/982 | 44.0% | 337/982 | 34.3% | 279/728 | 38.3% |
|  | 3-4 | 194/982 | 19.8% | 319/982 | 32.5% | 151/728 | 20.7% |
|  | 5-6 | 66/982 | 6.7% | 155/982 | 15.8% | 38/728 | 5.2% |
|  | 7+ | 23/982 | 2.3% | 65/982 | 6.6% | 3/728 | 0.4% |

**Table S3: Hospital (re)admissions by patient demographics and NEWS2 values**

|  | | | **N** | **No hospital (re)admission**  **(n=2,356)** | | **Hospital (re)admission**  **(n=177)** | |
| --- | --- | --- | --- | --- | --- | --- | --- |
|  | | |  | **n** | **%** | **n** | **%** |
| **Age (median, IQR):** | | | 2,533 | 73 | (59, 82) | 74 | (62, 83) |
|  | | 16-39 years | 184 | 174 | 94.6% | 10 | 5.4% |
|  | | 40-49 years | 164 | 157 | 95.7% | 7 | 4.3% |
|  | | 50-59 years | 280 | 262 | 93.6% | 18 | 6.4% |
|  | | 60-69 years | 432 | 392 | 90.7% | 40 | 9.3% |
|  | | 70-79 years | 677 | 641 | 94.7% | 36 | 5.3% |
|  | | 80+years | 796 | 730 | 91.7% | 66 | 8.3% |
| **Sex:** | | |  |  |  |  |  |
|  | | Male | 1,142 | 1,055 | 92.4% | 87 | 7.6% |
|  | | Female | 1,391 | 1,301 | 93.5% | 90 | 6.5% |
| **Source of referral:** | | |  |  |  |  |  |
|  | | Acute Hospital | 2,202 | 2,053 | 93.2% | 149 | 6.8% |
|  | | Ambulance Service | 34 | 30 | 88.2% | 4 | 11.8% |
|  | | Community Health Service | 176 | 162 | 92.0% | 14 | 8.0% |
|  | | Emergency Care Department | 1 | 1 | 100.0% | 0 | 0.0% |
|  | | GP Practice | 119 | 109 | 91.6% | 10 | 8.4% |
|  | | Not known | 1 | 1 | 100.0% | 0 | 0.0% |
| **Pathway*:** | | |  |  |  |  |  |
|  | | Respiratory | 1,040 | 992 | 95.4% | 48 | 4.6% |
|  | | Frailty | 446 | 404 | 90.6% | 42 | 9.4% |
|  | | OPAT | 483 | 449 | 93.0% | 34 | 7.0% |
|  | | Heart Failure | 178 | 155 | 87.1% | 23 | 12.9% |
|  | | General | 453 | 415 | 91.6% | 38 | 8.4% |
| **VW length of stay (days; median, IQR)** | | | 2,533 | 10 | (6, 14) | 8 | (5, 14) |
| **First NEW2 value** | | |  |  |  |  |  |
|  | 0 | | 600 | 569 | 94.8% | 31 | 5.2% |
|  | 1-2 | | 1,051 | 984 | 93.6% | 67 | 6.4% |
|  | 3-4 | | 569 | 525 | 92.3% | 44 | 7.7% |
|  | 5-6 | | 204 | 187 | 91.7% | 17 | 8.3% |
|  | 7+ | | 55 | 48 | 87.3% | 7 | 12.7% |
| **Max NEWS2 value** | | |  |  |  |  |  |
|  | 0 | | 115 | 107 | 93.0% | 8 | 7.0% |
|  | 1-2 | | 685 | 647 | 94.5% | 38 | 5.5% |
|  | 3-4 | | 887 | 833 | 93.9% | 54 | 6.1% |
|  | 5-6 | | 568 | 524 | 92.3% | 44 | 7.7% |
|  | 7+ | | 224 | 202 | 90.2% | 22 | 9.8% |

*Patients may be on more than one pathway during the same admission.

VW=Virtual ward. IQR=Interquartile range. OPAT=Outpatient Parenteral Antimicrobial Therapy.

Note. Percentages are row percentages rather than column percentages.

**Figure S1: Distribution of first, maximum and deterioration in NEWS2 values by sex**

**Figure S2: Distribution of first, maximum and deterioration in NEWS2 values by age**

**Figure S3: Distribution of first, maximum and deterioration in NEWS2 values by pathway**
