# Supplementary material for: Clinical acuity and National Early Warning Scores (NEWS2) of remotely monitored patients on virtual wards: A retrospective cohort study
Source: PLoS One. 2026 Apr 28;21(4):e0347678. doi: 10.1371/journal.pone.0347678 (PMC13123966; doi:10.1371/journal.pone.0347678)
Supplement: Attachment — Submitted filename: Response to Reviewers.docx [file pone.0347678.s004.docx]

| **Review comment** | **Response** | **Action** |
| --- | --- | --- |
| Reviewer #2: |  |  |
| Rather than putting the median and IQR by demographics in the supplementary material the authors could have actually shown the distributions through histograms. That would have been more informative. | Thank you for this suggestion. We have replaced supplemental table 2 with supplemental figures instead. | Supplemental figures S1-S3 added. |
| I have concerns about the large amount of missing data. The authors' response indicates that data at the beginning was unavailable because the full digitization had not been completed. If this is the case, I'm curious why then the study period was not adjusted to have only the time of full digitization? The authors are sort of mixing the study because they have patients that they describe for all variables and then some for only some variables. I do not quite feel the response to the reviewer's comment is satisfactory on that. | We felt it was important to include all available data to be able to fully describe the service and how it had progressed over time. However, following feedback from both reviewers we have now removed data from the first 9 months where respiratory rate was poorly recorded, and started our study period from October 2023. | We have removed data from Jan-Sep 2023 as suggested. |
| Reviewer #3: |  |  |
| I have four major issues with the paper: |  |  |
| 1.Missing data The levels of missing data are, as described by Reviewer 1, almost insurmountable in terms of reliability of the findings. This is not least as there are clear imbalances in the degree of missingness, for example 23% of the youngest patients are excluded rising to 60% of the oldest patients, who are not only the largest age group but surely the group to most likely experience the outcomes of interest.  The authors give at least two explanations for this. They state that as a new service more data were missing at the earlier stages of the service but there is no clear evidence of any improvement over time in Figure 1 that suggest limiting the scope to a more recent period would improve the reliability of the analysis. | Thank you for highlighting this.  As stated above, we have now excluded data from Jan-Sep 2023 to overcome this issue of poorly recorded respiratory rate.  To address the second issue regarding missing data due to provision of Doccla equipment, we have now reframed the paper to highlight that the focus here is people on virtual wards specifically who are remotely monitored. In the main paper, beyond the first paragraph of the results, we have therefore only included patients who recorded at least one observation on Doccla.  In addition, we have added in Supplemental Table 1 and some text at the beginning of the results section which describes the differences between patients with and without observations. We have also expanded the discussion around this. | Paper reframed, title updated, supplemental Table 1 added, discussion expanded. |
| In the discussion its stated that the kit is not universally distributed and in fact the elderly (and likely more frail) are less likely to get it. The flip side of this then is surely that those at highest risk of the outcome were systematically excluded from this dataset and therefore this dataset is more representative of the lower risk patients than the entire virtual ward population as a whole.  At the least the paper needs to make the degree of missingness in all groups explicit in a table and beef up the discussion of the implications of this. | Thank you for highlighting this. You are right that the population included in this paper are only those who are remotely monitored which appears to be more often those who are younger/ less frail.  We have now made this clearer in the paper and added supplemental Table 1 to address this. | Clarity added to methods and discussion around the focus on remotely monitored patients (who are often the younger/ potentially less sick patients).  Supplemental table 1 added to make the missingness in different groups more explicit. |
| 2.Only counting first admission The rationale for, and implications of, using only the first virtual ward admission where there was more than one is not made clear and appears to create a number of important gaps in the data which aren’t mentioned, quantified, or addressed.  For example in this dataset, no patient can have both outcomes, they cannot both be re(admitted) to hospital and die as an outcome of their first virtual ward admission.  In reality of course a patient could have both, i.e. they get (re)admitted and die in hospital, but here only the readmission counts.  Another scenario which appears to be overlooked is where a virtual ward admission leads to a hospital (re)admission which leads to another virtual ward admission. The authors wording implies that this second virtual ward admission is not in the dataset and so any readmission or death at the end of that is not counted.  Whilst counting these deaths would be addressing a subtly different question (akin to the outcomes of a spell of care) it’s hard to see why that wouldn’t be a more important question than one which essentially ignores anything that happens after the first virtual ward admission.  As it is the analysis takes a person based approach, nobody can appear in the dataset more than once. And to facilitate that they use only the first virtual ward admission. It is not clear why the analysis of virtual ward admission outcomes could not be on an virtual ward admission basis, i.e. individuals could appear more than once.  Or with perhaps a stronger rationale than the first admission, to be based on the final virtual ward admission within a continuous spell, mirroring the approach used for hospital episodes within a spell. At least this would tell you the ultimate outcome of the spell of care, rather than just of part of it.  If the paper remains focused on the first virtual ward admission then it needs to quantify somewhere the outcomes for the omitted ward admissions, principally the final virtual ward admission. This would then enable the reader to understand if the first admission approach was suitable or not. | We went round in circles about this and in the end chose to only include the first admission for each patient so as not to ‘double count’ patient demographics etc for those with more than one admission.  However, we agree with your comment that this results in potentially excluding outcomes for patients who have been admitted multiple times and ultimately died on a later virtual ward admission.  In light of your comments and following further discussion, we agree that taking an admissions-based approach rather than a person-based approach is preferable and enables us to include all outcomes. | Data and analyses rerun to include all admissions.  Text, tables and figures have been amended and updated accordingly. |
| 3.Composite outcome measure The composite outcome measure of (re)admission or mortality (on the virtual ward) is problematic as clearly the implications to the two aspects are markedly different.  This exact combination of mortality or hospital admission is criticised in a BMJ paper on the deficiencies in composite outcome measures where the components are “not of similar importance” (doi: <https://doi.org/10.1136/bmj.c3920>).  This is further compounded by the decision to count only the first virtual ward admission as discussed above. Death, the most important outcome to the patient, is certain to be undercounted in the sense of final outcome at the end of the connected spell of care.  Whilst the stated number of deaths is small, this is certain to be an underestimate and it would be better to treat the two outcomes separately, having first identified the final outcome of the spell. | We had originally planned to explore hospital (re)admissions and mortality separately, but due to the numbers of deaths being so small we chose to combine them.  However, we appreciate that this composite outcome is not ideal. As such, we have rerun the analyses for hospital (re)admission alone, and present mortality purely descriptively. | Analyses now presented with mortality and hospital (re)admission as separate outcomes. |
| At the very least the paper should tabulate somewhere the number of outcomes by the demographic groups to give some sense of the risk and therefore help assess the impacts of the inherent biases in the dataset.  Ideally it would establish and focus on the final outcome of the “spell” of care. | Thank you for this suggestion. We have added a supplemental table S3 of hospital (re)admissions by patient demographics and NEW2 values. We have not included a similar table for mortality due to small number and therefore disclosure risks. | Supplemental Table S3 added. |
| 4.Unadjusted analysis  I don’t really understand why just univariate analysis has been used.  It would seem that a multivariable approach would be more informative and may indicate important patterns not visible at the moment, for example perhaps the score is predictive in certain groups (perhaps those with less missing data) and not in others.  At the very least there needs to be a clear rationale stated as to why the current univariate analytical approach is the best approach to assessing predictiveness and give reassurance that there are no subgroup effects that have been overlooked. | While we agree that unadjusted analyses may mask some patterns in specific groups, we have chosen to keep the analyses unadjusted for two reasons: first, the number of outcomes is reasonably small and further cutting this up is likely to make the results uninterpretable. Secondly, the aim of this paper was to look at the predictive ability of NEWS2 across all remotely monitored virtual ward patients, not in specific subgroups.  However, we appreciate that the breakdown of outcomes by groups may be of interest to some readers, so we hope that the addition of supplemental table S3, as suggested, provides this additional information. | Supplemental Table S3 added to describe outcomes by patient demographics. |
