# Supplementary material for: Clinical acuity and National Early Warning Scores (NEWS2) of remotely monitored patients on virtual wards: A retrospective cohort study
Source: PLoS One. 2026 Apr 28;21(4):e0347678. doi: 10.1371/journal.pone.0347678 (PMC13123966; doi:10.1371/journal.pone.0347678)
Supplement: Attachment — Submitted filename: Responses to reviewer comments v1.0.docx [file pone.0347678.s003.docx]

| **Editor comments** | **Response** | **Action** |
| --- | --- | --- |
| 1. When submitting your revision, we need you to address these additional requirements.    Please ensure that your manuscript meets PLOS ONE's style requirements, including those for file naming. The PLOS ONE style templates can be found at xxx. |  | This has now been done. |
| 2. We note that the grant information you provided in the ‘Funding Information’ and ‘Financial Disclosure’ sections do not match.    When you resubmit, please ensure that you provide the correct grant numbers for the awards you received for your study in the ‘Funding Information’ section. |  | The funding information on the online form has now been updated to reflect the funding statement in the manuscript. |
| 3. Thank you for stating in your Funding Statement:  This project was funded by NHS England South West and supported by the National Institute for Health and Care Research Applied Research Collaboration West (NIHR ARC West). The views expressed in this article are those of the authors and not necessarily those of NHS England, NHS Improvement, the NIHR or the Department of Health and Social Care.    Please provide an amended statement that declares *all* the funding or sources of support (whether external or internal to your organization) received during this study, as detailed online in our guide for authors at<http://journals.plos.org/plosone/s/submit-now>.  Please also include the statement “There was no additional external funding received for this study.” in your updated Funding Statement.  Please include your amended Funding Statement within your cover letter. We will change the online submission form on your behalf. |  | The funding information on the online form has now been updated to reflect the funding statement in the manuscript.  The statement “There was no additional external funding received for this study.” Has also been added to the end of the funding statement in the manuscript. This is also detailed in the cover letter. |
| 4. We note that you have indicated that there are restrictions to data sharing for this study. For studies involving human research participant data or other sensitive data, we encourage authors to share de-identified or anonymized data. However, when data cannot be publicly shared for ethical reasons, we allow authors to make their data sets available upon request. For information on unacceptable data access restrictions, please see<http://journals.plos.org/plosone/s/data-availability#loc-unacceptable-data-access-restrictions>.    Before we proceed with your manuscript, please address the following prompts:    a) If there are ethical or legal restrictions on sharing a de-identified data set, please explain them in detail (e.g., data contain potentially identifying or sensitive patient information, data are owned by a third-party organization, etc.) and who has imposed them (e.g., a Research Ethics Committee or Institutional Review Board, etc.). Please also provide contact information for a data access committee, ethics committee, or other institutional body to which data requests may be sent.    b) If there are no restrictions, please upload the minimal anonymized data set necessary to replicate your study findings to a stable, public repository and provide us with the relevant URLs, DOIs, or accession numbers. Please see<http://www.bmj.com/content/340/bmj.c181.long> for guidelines on how to de-identify and prepare clinical data for publication. For a list of recommended repositories, please see <https://journals.plos.org/plosone/s/recommended-repositories>. You also have the option of uploading the data as Supporting Information files, but we would recommend depositing data directly to a data repository if possible.    Please update your Data Availability statement in the submission form accordingly. | The data is owned by a third party, Sirona Care & Health, and was obtained during routine patient monitoring.  We obtained ethical approval to use this data for the purpose of the study, but as patients did not provide consent, it is not appropriate to publicly share their data.  For transparency, we have uploaded our Stata analysis code to GitHub so that if someone was to obtain the data from Sirona themselves, they would be able to replicate what we did. | We have amended our data statement to clarify this, added details of the GitHub repo where our code can be accessed, and have also added a contact email address for Sirona Care & Health who would need to be contacted for the data. |
| 5. For studies involving third-party data, we encourage authors to share any data specific to their analyses that they can legally distribute. PLOS recognizes, however, that authors may be using third-party data they do not have the rights to share. When third-party data cannot be publicly shared, authors must provide all information necessary for interested researchers to apply to gain access to the data. (<https://journals.plos.org/plosone/s/data-availability#loc-acceptable-data-access-restrictions>)    For any third-party data that the authors cannot legally distribute, they should include the following information in their Data Availability Statement upon submission:  a) A description of the data set and the third-party source  b) If applicable, verification of permission to use the data set  c) Confirmation of whether the authors received any special privileges in accessing the data that other researchers would not have  d) All necessary contact information others would need to apply to gain access to the data |  | We have updated our data statement accordingly. |
| 6. Your ethics statement should only appear in the Methods section of your manuscript. If your ethics statement is written in any section besides the Methods, please move it to the Methods section and delete it from any other section. Please ensure that your ethics statement is included in your manuscript, as the ethics statement entered into the online submission form will not be published alongside your manuscript. |  | The ethics statement has been removed from the additional details at the end of the manuscript. |
| 7. We note that you have referenced (Lyndon H, Viney T.) which has currently not yet been accepted for publication. Please remove this from your References and amend this to state in the body of your manuscript: (“Lyndon H, Viney T. [Submitted]”) as detailed online in our guide for authors <http://journals.plos.org/plosone/s/submission-guidelines#loc-reference-style> | This paper has been accepted and published since our manuscript submission. | The published version of this paper has now been referenced. |
| 8. Please include captions for your Supporting Information files at the end of your manuscript, and update any in-text citations to match accordingly. Please see our Supporting Information guidelines for more information:<http://journals.plos.org/plosone/s/supporting-information>. |  | These have now been added at the beginning of the additional information after the references. |
| 9. If the reviewer comments include a recommendation to cite specific previously published works, please review and evaluate these publications to determine whether they are relevant and should be cited. There is no requirement to cite these works unless the editor has indicated otherwise. |  | n/a |
| Additional Editor Comments:  The reviewers have raised major concerns about data accuracy and comparison to between virtual ward and acute wards It has been decided that the authors will be granted a second chance to improve the manuscript provided that the authors will address those major concerns | We hope our responses below and corresponding edits to the manuscript have addressed these concerns. |  |

| **Reviewer comments** | **Response** | **Action** |
| --- | --- | --- |
| The authors conduct a retrospective analysis of physiological parameters for patients managed on a virtual ward network.  There are a number of areas of major concerns which need to be considered. | Thank you for your helpful comments. Please see responses to all issues raised below. |  |
| The first area is the definition and description of the hospital level interventions delivered on this virtual ward network. This is missing and it is essential for interpretation of the manuscript. Was their point of care ultrasound delivered diagnostics, point of care blood monitoring, intravenous therapies etc.. Delivering physiological monitoring in isolation is insufficient to meet this criteria. Further why have OPAT services been included? OPAT services has been in existence since the last 1990s and should not be regarded as meeting the criteria for virtual ward admission unless further interventions are also being delivered in the service. | Thank you for highlighting the missing details about the virtual wards services. We have added more detail about the BNSSG virtual wards in the introduction.  BNSSG did not have an OPAT service before the virtual ward program began. As such, in this region in sits within virtual ward care. While this group does include people who are relatively well but require antibiotics, it also includes people who are acutely unwell and need additional care and monitoring in addition to antibiotics. There is no way to separate these two groups so for completeness, we chose to include them in our analyses. | The following text has been added to the introduction:  “As well as monitoring of physiological observations, patients on these wards receive clinical care including taking blood, urine and sputum specimens, dressing changes, delivering of intravenous medications including diuretics and antibiotics. Many patients are visited regularly, while others are monitored remotely and may only be seen face-to-face if their observations or pathway symptoms specific questionnaire completion suggest intervention might be required.” |
| The second area that needs consideration is the equivalence in the paper given to clinical acuity and physiological stability (as measured by NEWS2). It does not appear that any other markers of clinical acuity are examined in the paper and by themselves physiological parameters are not a good measure of clinical acuity. | We believe that as a track and trigger system, NEWS2 in a good measure of both clinical acuity and physiological stability. One off measure can be used as a measure of clinical acuity (“trigger”), but multiple measures over time also provide an understanding of the patients physiological stability (“track”).  Further, many other publications have used NEWS2 as a marker of clinical acuity. For example, the aim of the SAMBA report is to “describe the severity of illness of acute medical patients presenting to Acute Medicine, the speed of their assessment, their pathway and progress at seven days after admission and to provide a comparison for each participating unit with the national average (or ‘benchmark’).” They have used NEWS2 to assess many of these markers.  However, we appreciate that there are other factors involved in defining clinical acuity (such as blood markers and clinical judgement), which are measured on the virtual wards but are not presented in this paper because they are not part of the remote monitoring protocol. We have therefore added a sentence towards the end of our introduction acknowledging this and explaining that for the purpose of this paper we are using NEWS2 as a measure of clinical acuity. | We have added the following sentence towards the end of the introduction:  “While clinical observations alone cannot provide the full picture of clinical acuity, NEWS2 is a strong indicator of acute clinical illness and deterioration [20], and can be monitored remotely, and as such is used to represent clinical acuity in this paper.” |
| The third area is the NEWS2 data itself:- |  |  |
| 1. Only 57% of patients have a NEWS2 score that can be calculated. This is an almost insurmountable issue and the conclusions made in the paper should be stated with significantly less confidence in the context of this missing data. | This paper includes patients from day one of these virtual wards existing. As such, the full digitalisation of the wards was not complete at this time. Some patients who were visited in person had their observations recorded on paper rather than digitally, so these were no accessible to us for this evaluation. This is a very common issue at the beginning of a new service when it is still evolving and working through teething problems. This is not the case on these wards any more as even those who are not using the Doccla equipment still have their observations updated to EMIS digitally.  However, you are right that this means the data might not be representative of all patients on these virtual wards. We have already highlighted this in the limitations section, but we have added an additional sentence making it clearer than this issue might result in the data not being representative.  Further, we have added a caveat to the statement in the final paragraph about the similarities between virtual wards patients and those admitted to acute medical units. | Clarifying statements have been added to the limitations section and the implications for research section in the discussion. |
| 2. How did the authors derive their implausible physiological parameters to be excluded? How many of these were there? This raises concerns about the accuracy of the device used. How many patients had a respiratory rate of >50 for example. Further, when do the authors think people may have a temperature >43? | Implausible values were decided based on patterns in the data and clinical judgement.  Following your comment we have reassessed these and agree that a temperature >43 is not plausible. In our data we only had one value between 40.1 and our previous cut-off of 45, which was 43.2. We agree that this is probably implausible and have removed this single observation from the analysis and updated the methods text and Table 2 accordingly (it didn’t affect any of the NEWS2 scoring).  These implausible values were unlikely to be related to the equipment used but simply data entry errors (including typos and entering values in the wrong box - for example entering a blood pressure in the respiratory rate box). The total number of excluded values from all fields was 462/155,759 (0.3%) observations. | We have clarified how we derived the implausible values in the methods and have also added a paragraph about these implausible values to the limitations section in the discussion:  “We identified some implausible values in the clinical observations (462/155,759 observations [0.3%]) which we chose to exclude for the purpose of analysis. Data entry errors of this magnitude are expected in this type of data collection. In terms of the actual service, recording of these extremely high or low values would trigger a phone call to the patient from the clinical team to check how they were feeling, alongside a review of symptoms and repetition of the observations whilst on the telephone. The error would be promptly identified, and no further action would be needed.” |
| 3. "Clinical acuity" cannot be compared to acute hospital care when 12% of initial observations were taken >48 hours after "admission" to the virtual ward. | We agree that 12% of first digitally recorded observations being >48 hours after admission to virtual wards is not ideal, but there will also be patients in the SAMBA report who had their observations recorded late, or who did not have a NEWS2 value recorded.  You are right that we aren’t comparing like for like, but given that SAMBA is a well-respected acute medicine audit, and this is one of the first papers to explore physiological observations in virtual wards patients, we believe this is still a useful comparison. | We have added that 12% of first scores were more than 2 days after admission to the limitations section. |
| The fourth area is comparison to hospital care:- |  |  |
| 1. Over 80% of the patients have undergone a period of acute inpatient care, assessment and treatment prior to discharge to a "virtual ward". Therefore, this is an entirely different cohort to those analyzed during SAMBA. Their clinical acuity will have been assessed as less than those requiring inpatient care by a senior clinician. | As stated above, you are right that we aren’t comparing like for like. However, understanding the similarities in physiology between this virtual ward cohort and those admitted to acute medical care is important.  We are arguing that while these patients have been assessed to be safely managed at home, they do in fact still have a similar acuity to those treated in hospital. The difference being these patients are likely to be more stable than those being kept in hospital. | We have moved the discussion of the SAMBA report to the comparison with other literature section rather than summary of findings. We have also added discussion of the physiological stability of virtual ward patients and highlighted that perhaps this is a key difference between them and long-term hospital patients. |
| 2. The prediction in this cohort is thus predominantly not "admission" to hospital but actually a marker of "readmission". This should be the metric in this study. | Thank you for highlighting this. We have changed “admitted” to “(re)admitted” throughout the paper. | We have changed “admitted” to “(re)admitted” and “admission” to “(re)admission” throughout, including in figure headings. We have explained this term in the statistical analysis section. |
| 3. The conclusion that the clinical acuity of those admitted to virtual wards is similar to those admitted on the acute medical take is thus deeply flawed. | We have changed the opening sentence of the conclusion to state that the NEWS2 values of our virtual ward patients are similar to those admitted to acute medical wards (rather than the clinical acuity being similar). We have also clarified in this sentence that these findings are based only on patients with digitally recorded clinical observations. | “The distribution of NEWS2 values of patients admitted to virtual wards (who had their clinical observations digitally recorded) is similar to that of patients admitted to acute medical wards.” |
