# Supplementary material for: Clinical acuity and National Early Warning Scores (NEWS2) of remotely monitored patients on virtual wards: A retrospective cohort study
Source: PLoS One. 2026 Apr 28;21(4):e0347678. doi: 10.1371/journal.pone.0347678 (PMC13123966; doi:10.1371/journal.pone.0347678)
Supplement: Attachment — Submitted filename: Response to reviewer comments.docx [file pone.0347678.s005.docx]

Reviewer #3: Thank you for your consideration of my comments and for the resulting changes you have made which make it generally much easier to read and interpret.

My only final comment is that you can see now in the new supplemental table 3 a gradient for hospital readmission risk by First NEWS2 score (NEWS2 0 = 5.2%, then 6.4%, 7.7%, 8.3% up to NEWS2 7+ = 12.7%), which surprisingly you don't mention in the actual paper and seems at face value to to run contrary to your claim that "...prediction of readmission to hospital using NEWS2 in this setting was poor".

Plugging those numbers into a quick Chi square confirms your finding that its not statistically significant but it does to me seem to warrant a mention. If your study was twice the size then the Chi square would be highly significant (and clinically meaningfully so as the risk in the 7+ group is 2.5 times that of the risk the 0 group).

It does seem to me that your paper would benefit from being a bit clearer about this as a weakness (there is evidence of a trend but the sample size is relatively small) rather than just saying its prediction ability was poor.

Response: Thank you for reassessing our manuscript. We are pleased the previous amendments have made the paper much easier to read and interpret. As requested, we have made an additional amendment to present the percentage of (re)admitted patients by NEWS2 value in the results section, and have also added a sentence in the discussion highlighting the potential power issues due to small numbers. We have not amended the conclusion as we had already caveated the findings with the sentence “However, hospital (re)admission rates were low (7%) so this should be interpreted with caution.”
